# Progress on wearable triboelectric nanogenerators in shapes of fiber, yarn, and textile

**DOI:** 10.1080/14686996.2019.1650396

**Published:** 2019-07-31

**Authors:** Jiaqing Xiong, Pooi See Lee

**Affiliations:** School of Materials Science and Engineering, Nanyang Technological University, Singapore, Singapore

**Keywords:** Triboelectric nanogenerator, fiber, yarn, fabric, textile, 50 Energy Materials, 206 Energy conversion / transport / storage / recovery

## Abstract

Textile has been known for thousands of years for its ease of use, comfort, and wear resistance, which resulted in a wide range of applications in garments and industry. More recently, textile emerges as a promising substrate for self-powered wearable power sources that are desired in wearable electronics. Important progress has been attained in the exploitation of wearable triboelectric nanogenerators (TENGs) in shapes of fiber, yarn, and textile. Along with the effective integration of other devices such as supercapacitor, lithium battery, and solar cell, their feasibility for realizing self-charging wearable systems has been proven. In this review, according to the manufacturing process of traditional textiles starting from fibers, twisting into yarns, and weaving into textiles, we summarize the progress on wearable TENGs in shapes of fiber, yarn, and textile. We explicitly discuss the design strategies, configurations, working mechanism, performances, and compare the merits of each type of TENGs. Finally, we present the perspectives, existing challenges and possible routes for future design and development of triboelectric textiles.

## Graphical Abstract

**Figure:**
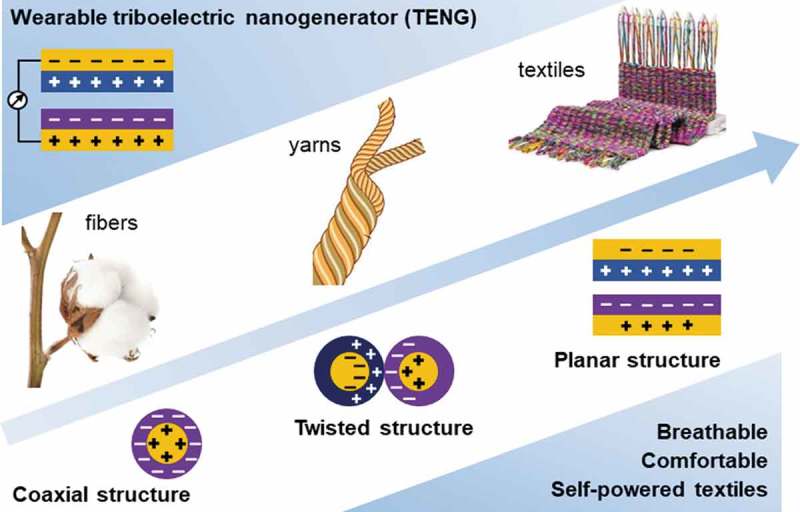

## 1. Introduction

Portable and wearable electronics are emerging as indispensable requirements in modern living for communication, motion tracking, health monitoring, and wearable displays [1–6]. In terms of the ultimate demands, the electronic modules for the next-generation wearable electronics are required to be lightweight, flexible, breathable, washable and durable to meet the practicality and aesthetics, allowing the maximum degree of freedom for movements and maximum comfort [7–9]. Accordingly, better wearability of the energy modules is required in terms of conformability and comfort in order to be acceptable for wearable electronics [10]. Triboelectric nanogenerator (TENG), which couples triboelectrification and electrostatic induction to convert mechanical energy into electricity, can harvest electricity from the ambient mechanical motions to drive the electronics and realize the self-powered wearable systems [11–14].

TENG operation is based on the coupling effect of triboelectric and electrostatic induction. The working mechanisms of the most common mode of vertical contact-separation can be illustrated as follows [12]. TENG is composed of two materials (at least one of them is insulator) with back electrodes separated by elastic spacer. When an external force is applied to contact the two triboelectric materials, triboelectric charges would be induced and maintained on the surfaces of two materials. When the mechanical force is released, the two materials would separate to create a potential difference between the back electrode and the contact object, driving electrons through the external circuit to screen the induced triboelectric charges, therefore generating a current pulse. Subsequently, when an external force once again reduces the separation, an electric potential difference with reversed polarity is produced, resulting in electrons flow in a reversed direction, keeping the screening inductive charges on the back electrode until a contact is applied again. When periodical force is applied, continuous electric pulses would be generated as output voltage and current. Triboelectric output depends on the relative position of the two materials in the triboelectric series, which would contribute different polarities for electrostatic induction, resulting in different power densities [15]. In addition, triboelectric output also relies on the surface structure of materials, higher surface roughness could contribute stronger frictional effect, producing enhanced electricity outputs [16–24]. Various TENGs have been explored based on different substrates, triboelectric materials, electrodes and spacers [25,26]. Polymer films are the commonly used triboelectric materials owing to they are lightweight, flexible, and robust, resulting in good triboelectric output [27–31]. However, they may not be the best choices for wearable TENGs in terms of breathability, comfortability, and wearability.

Textile provides intrinsic porous structure and high surface roughness, traditionally used for protection, warming, and aesthetics [32]. In line with the rapid growth in modern portable and wearable electronics, realizing textiles with additional functions is of great significance, such as electricity generation, energy storage, and color/thermal management, which could endow textiles with new vitality for smart wearable systems [8,10,33]. Self-powered textile is one of the fundamental needs to attain the self-charging wearable systems. It is realizable by triboelectric effect to convert the mechanical energy of movements or biomechanical energy of motions into electricity, promising to continuously power up the other electronic modules. Important progress has been achieved on exploring the configuration of textile TENG, improving the triboelectric performance, and considering washability and comfortability of devices [34–37].

The manufacturing process of the traditional fabrics and textiles starts from fibers, twisting into yarns, and sewing/weaving into textiles, followed by functional finishing to produce the clothes/garments for daily use. Accordingly, we summarize the progress on wearable TENGs based on the different carriers and configurations (one dimension (1D), and two dimensions (2D)) of devices, including design strategies, fabrication of TENGs and hybrid devices based on fibers, yarns, and fabrics/textiles. We illustrate the advantages and disadvantages of each type of TENGs, including their constructions, performances, and feasibilities for weaving, patterning, and washing. According to the processing requirements for traditional textiles, we propose reliable strategies and routes to fabricate TENGs for different wearable applications, providing an effective reference for material choices and device configurations for wearable TENGs.

## 2. Carriers and fabrication routes of textile TENG

**Figure 1.**
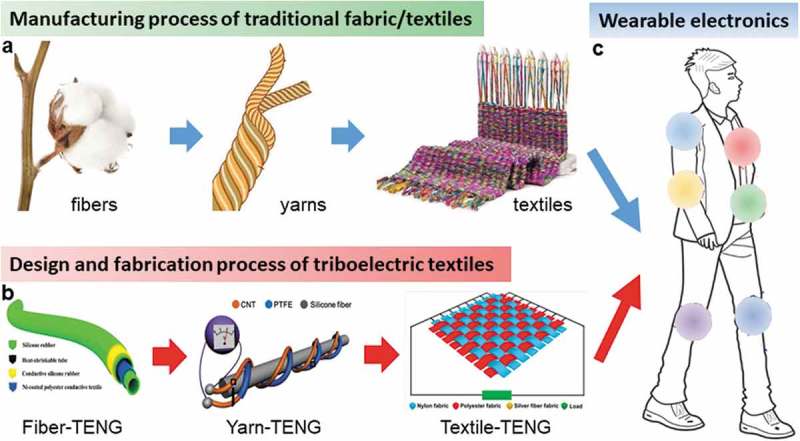
Carriers and routes for fabrication of wearable TENGs. (a) Manufacturing process of the traditional fabric/textile. (b) Design and fabrication process of the textile triboelectric nanogenerator (textile TENG). Reproduced with permission from [40,56,67]. Reproduced under the terms of the Creative Commons Attribution 3.0 International License (CC BY-3.0) (https://creativecommons.org/licenses/by/3.0/) [40]. Copyright 2018, The Authors, published by Royal Society of Chemistry. Copyright 2015 [56], John Wiley and Sons; Copyright 2014 [67], American Chemical Society. (c) Schematic illustration of textile TENGs that can be located at various positions to serve as self-powered wearable systems. Reproduced under the terms of the Creative Commons Attribution 4.0 International License (CC BY-4.0) (https://creativecommons.org/licenses/by/4.0/) [86]. Copyright 2018, The Authors, published by Springer Nature.

Conventional fabrics and textiles are manufactured starting from the natural or synthetic fibers, followed by twisting and spinning into yarns with varying dimensions for further weaving/knitting into textiles; thereafter, functional textiles and clothes can be attained by desizing, bleaching, dyeing, and finishing [32]. Accordingly, electronic textiles can be also realized based on those carriers (fibers, yarns, fabrics, and textiles) via the same process (Figure 1). Various electronic devices have been realized in shapes of fibers, yarns, and textile to confirm the feasibility [8,35,38,39]. To consider the energy-generating efficiency and practicability, wearable TENGs based on fibers, yarns, and fabrics would be ultimately operated in the form of fabric or textile, delivering better wearability and enhancing ease of incorporation with garments.

Considering the precise structure-design and pattern diversity of triboelectric textile, TENGs based on fibers and yarns are important units for the textile devices, each fiber or yarn can be designed into the individual coaxial or twisted 1D TENG. Although high triboelectric outputs are hard to be attained on a single fiber/yarn device, the 1D TENG can be further fabricated into textile TENGs via knitting/weaving for scalable self-powered textiles with high electric outputs. Meanwhile, concerns of good mechanical property and processability of fiber/yarn TENGs need to be considered during the subsequent weaving process, such as high tensile strength, wear resistance, water resistance, and length of devices, which remain as the challenges for fiber/yarn-based TENGs.

On the other hand, 2D TENGs can be directly realized on the fabrics/textiles as planar single/multi-layered structures, while conductive or nonconductive yarns/textiles can serve as the electrodes and triboelectric materials. Besides the post sewing/weaving of 1D TENGs to fabricate textile TENGs, another way is to directly apply functional finishing onto the textiles. Accordingly, 2D devices of various sizes are readily attainable, promising for large-scale production. However, most of the reported textile TENGs are incorporated with polymer materials to realize triboelectric properties and good mechanical performances, it is well known that the polymers would easily block the porous structure of textile, often lead to poor breathability and low comfort level to the users. A daunting challenge remains in the fabrication of textile TENG without loss of the intrinsic merits of textile, such as breathability, affinity, and comfortability.

Here, we summarize the important works of TENGs based on fibers, yarns, and fabrics/textiles, respectively. We mainly focus on introducing the configurations of each type of device, and their working mechanisms as well as triboelectric performances. We compared the merits and shortcomings of those wearable TENGs based on different substrates, proposing effective ways for designing the scalable triboelectric textiles with better breathability and functionality. In addition, on the basis of fibers, yarns, and fabrics/textiles, self-charging hybrid devices incorporated with TENGs such as supercapacitor, battery, and solar cell have been summarized, showing the integration potential of textile TENGs for various self-powered systems, promising to the next-generation all-textile wearable electronics with better comfortability and practicability for our life.

## 3. Fiber-based TENGs

Fiber is the basic unit of textile, it is a flexible material with a large ratio of length to diameter that can be conductive or nonconductive. Usually, natural fibers could provide better affinity but relatively lower mechanical strength. Direct fabrication of TENGs on natural fibers remains a challenge because this substrate is ultrafine and flexible, making it hard to be processed and operated. So far, all the fiber TENGs are designed as a coaxial structure based on the insulating synthetic polymer fibers or conductive wires with a large diameter and operability [40–45]. Normally, nonconductive polymer fibers could serve as carrier, triboelectric materials, and encapsulation layers for triboelectric devices. Conductive wires such as fibers or synthetic conductive fibers would be used as electrodes in straight or coiled forms.

### 3.1. Coaxial fiber TENGs

**Figure 2.**
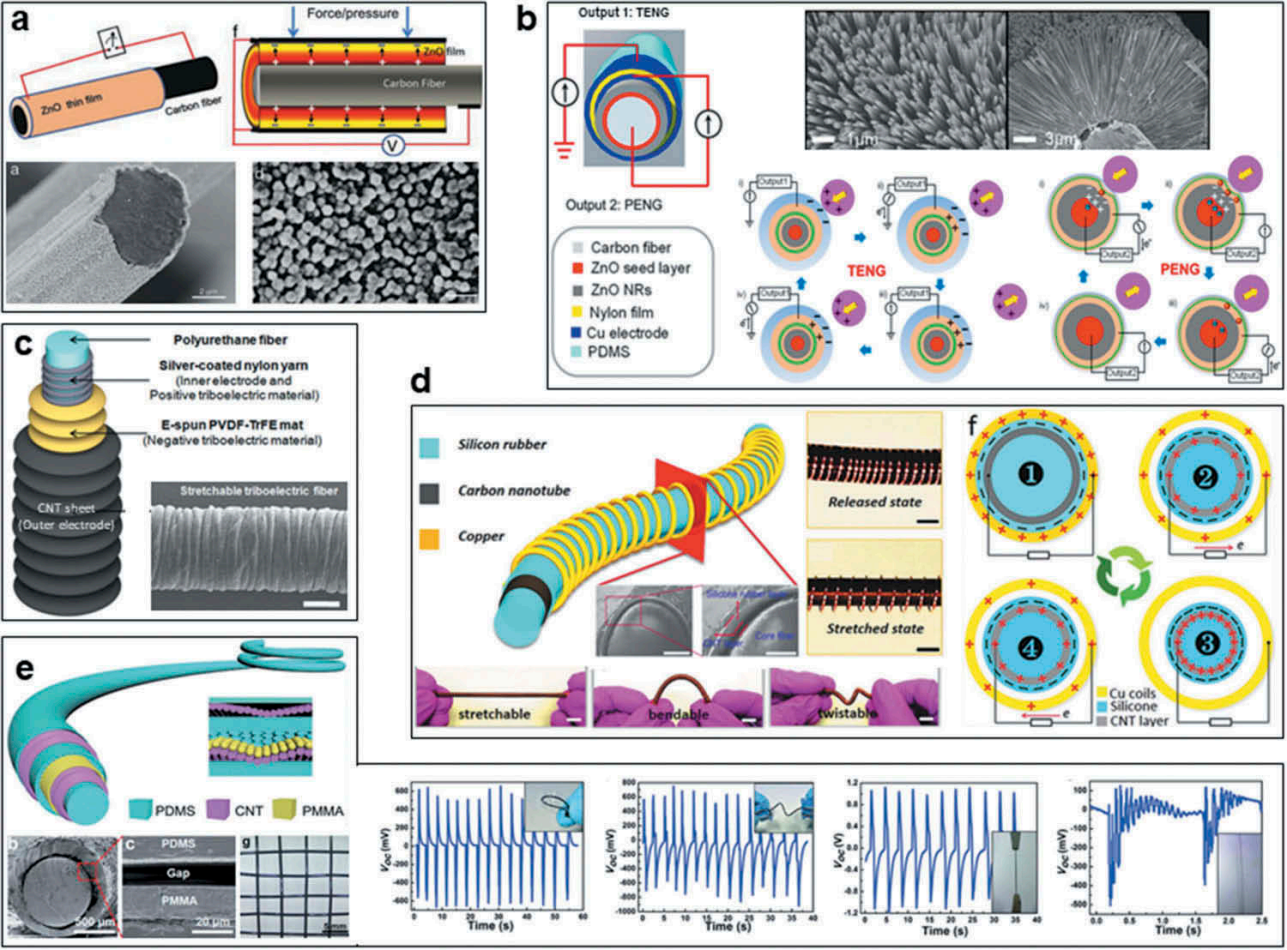
Fiber-based nanogenerators. (a) Carbon fiber piezoelectric nanogenerator (PENG). Reproduced with permission from [41]. Copyright 2015, John Wiley and Sons. (b) Flexible carbon fiber-based triboelectric nanogenerator (TENG). Reproduced with permission from [42]. Copyright 2014, American Chemical Society. (c) Stretchable TENG based on polyurethane (PU) fiber. Reproduced under the terms of the Creative Commons Attribution 4.0 International License (CC BY-4.0) (https://creativecommons.org/licenses/by/4.0/) [43]. Copyright 2016, The Authors, published by Springer Nature. (d) Stretchable TENG based on silicone fiber. Reproduced with permission from [44]. Copyright 2017, John Wiley and Sons. (e) Deformable TENG based on PDMS fiber. Reproduced with permission from [45]. Copyright 2017, Royal Society of Chemistry.

The first fiber nanogenerator was realized by Li and Wang using the piezoelectric effect in 2011 (Figure 2(a)) [41]. Textured zinc oxide (ZnO) thin films were cylindrically covered on the carbon fibers, the resultant piezoelectric nanogenerator (PENG) could generate an output voltage of 3.2 V and current density of 0.15 µA cm^−2^ under applied pressure, it could serve as a sensor in a non-contact mode by air pressure to monitor the heart rate of human.

In 2012 it was confirmed that triboelectric effect can convert mechanical energy into electricity [12]. After that tremendous efforts have been dedicated to design planar TENGs with multiple structures and improved performances based on various polymer substrates, some of them have been demonstrated for wearable applications [46–54]. The 1D wearable TENG in fiber shape was firstly explored in 2014 by Wang et al., a coaxial carbon fiber-based hybrid nanogenerator (diameter of 3 mm) combined with PENG (core) and TENG (shell) was developed based on both piezoelectric and triboelectric effects under mechanical contact and pressure (Figure 2(b)) [42]. The device can be woven into a smart cloth to harvest mechanical energy from human motions and serve as a self-powered strain sensor, the instantaneous output power density of TENG and PENG can achieve 42.6 and 10.2 mW/m^2^. In this case, vertical contact-separation friction between adjacent fibers is the driving force for both TENG and PENG, a fiber could induce opposite charges on the surface of adjacent fibers under interaction upon applying an external force, driving TENG to generate triboelectricity. Further pressing and releasing between the fibers could trigger the PENG to produce electricity by piezoelectric effect. This vertical contact-separation mode is the most common structure of triboelectric devices, which is an efficient working mode for fiber TENGs that have high chances to contact and separate each other during daily usage.

Besides the friction triggered by adjacent fibers, triboelectric effect could also occur in a coaxial fiber TENG upon stretching. Kim et al. designed a stretchable triboelectric fiber (~50% strain) by prestretching method for self-powered kinematic sensing textile in 2016 (Figure 2(c)), which generates electricity based on the reversible gap (repetitive friction) induced by the Poisson’s ratio difference between the core fiber (silver-coated nylon/polyurethane) and the shell (wrinkled electrospun film of polyvinylidene fluoride-co-trifluoroethylene (PVDF-TrFE)/carbon nanotube (CNT) layer enabled by pre-stretched core fiber) during tensile deformation [43]. The voltage increased with frequency from 9 mV (3 Hz) to 24 mV (10 Hz) at a strain of 50%, accordingly, current increased from 2 nA to 8 nA. The stretchable fiber TENG (~490 µm) can be incorporated into a commercial textile to detect magnitude and direction of human motion. To have better stretchability, stretchable electrode based on conductive ink of carbon nanotube (CNT)/Parafilm matrix was used by Wang et al. to dip-coat on a silicone rubber fiber (diameter 3.5 mm, length 10 cm), with coating of another silicone rubber layer as the insulating layer, the resultant fiber was convolved by Cu wires to assemble the fiber TENG (Figure 2(d)), which can be operated at high strain up to 70% due to the stretchable ink and coiled Cu electrode [44]. The output performance can be tuned by changing the number of coils, working frequency, and tensile strain. The maximum outputs can achieve 140 V and 0.18 µA cm^−1^ based on a device with a length of 22 cm. A broad range of applications have been demonstrated on this fiber TENG to power the commercial capacitor, LCD screen, digital watch/calculator, and self-powered acceleration sensor.

In practical applications, most of the external mechanical motions are not limited to vertical and axial deformations for triggering the fiber TENGs. To accommodate diverse mechanical stimuli such as pressing, bending, twisting, stretching and vibrating, a coaxial TENG for both energy harvesting and sensing under deformation was developed by Peng et al. in 2017, using aligned carbon nanotube sheets as inner and outer electrodes based on triboelectric polymers of polydimethylsiloxane (PDMS) and polymethyl methacrylate (PMMA) (Figure 2(e)). Rough polymer layers of PMMA microspheres and porous PDMS were realized to enhance the triboelectric effect, an air gap between two polymer layers was produced using sucrose as sacrificial template to ensure effective contact and separation between triboelectric layers [45]. The fiber TENG was flexible, stretchable, weavable, and adaptable to convert the multidirectional mechanical energy to electricity, the maximum outputs can achieve 5 V and 240 nA under compression force of 25 N, it was highly durable and could be integrated into other electronic devices as a multiple response sensor.

### 3.2 Potential and challenges of fiber TENGs

Various coaxial TENGs have been achieved to harvest multidirectional mechanical energy due to the excellent deformability of fiber materials, they can work individually or be incorporated/woven into textiles, serving as not only self-powered sources but also sensors for human motion and health monitoring. With regards to configuration, the fibers TENGs can be based on the core of electrode (carbon fiber) or triboelectric material (elastomer), accordingly, the shell could be triboelectric layer or electrode, the components are realized one by one by dip-coating and convolving process. In terms of triboelectric output, the power density of individual fiber device is too low to meet the requirements of powering wearable devices. From the aspect of wearability, current fiber TENGs consist of synthetic polymers and metal wire/fiber for triboelectric layers and electrodes; although they are weavable, their widespread integration with daily textiles is still poor due to large diameters (~3.5 mm) and insufficient lengths (~10 cm). The connection between the fiber TENGs during sewing and weaving would be also a concern that needs to be considered and addressed.

Designing TENGs on natural fibers is promising to realize the weavability and wearability of the devices. However, it remains a challenge to realize the fabrication process on natural fibers that are ultrafine and highly flexible. Furthermore, the subsequent weaving process would also be a formidable task due to the mechanical properties of those fiber TENGs. Therefore, the current coaxial fiber TENGs with visible and operational dimensions are effective to serve as individual device for energy harvesting and sensing in specific environments that require one-dimensional (1D) energy harvester.

## 4. Yarn-based TENGs

Yarns consist of interlocked fibers that are made up of two or more twisted fibers; they are suitable for the production of textiles, sewing, crocheting, knitting, weaving, embroidery or ropemaking [55]. Larger diameters endow it with better mechanical strength and processability, making it more suitable for construction of TENGs that require mutual friction of two objects. Compared with fiber TENG, yarn TENG offers more choices on fiber type and working mode, the contact-separation can be realized between different fibers under multiple deformations of device such as bending, twisting, stretching. In the following section, we summarize the configuration of yarn TENGs as well as their advantages and challenges in structure and performance [56–63]. Furthermore, we introduce the hybrid 1D devices for self-charging energy management based on TENGs of fibers and yarns [64–66].

### 4.1. Yarn TENGs

**Figure 3.**
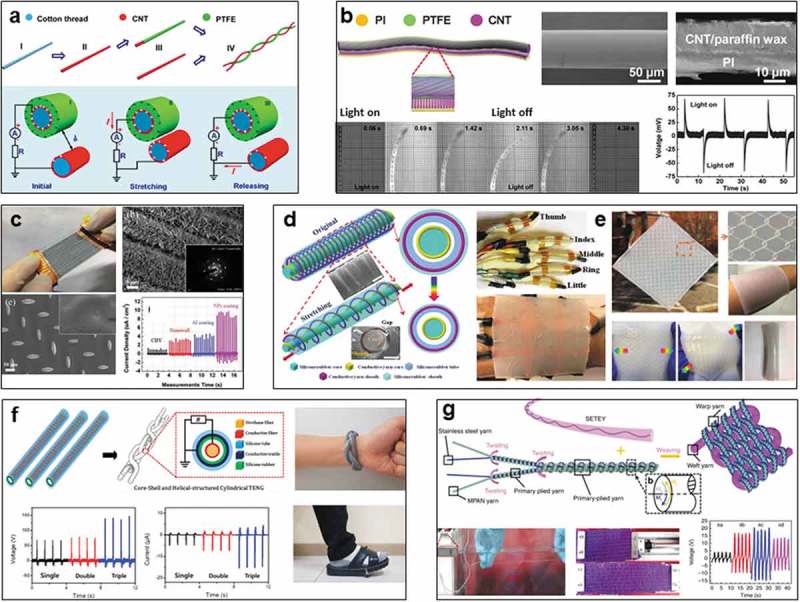
Yarn-based TENGs. (a) Cotton thread-based TENG. Reproduced with permission from [57]. Copyright 2014, American Chemical Society. (b) Photoelectric conversion CNT yarn TENG. Reproduced with permission from [58]. Copyright 2016, John Wiley and Sons. (c) Hierarchically nanostructured yarn TENG. Reproduced with permission from [59]. Copyright 2017, John Wiley and Sons. (d) Core-sheath yarn TENG. Reproduced with permission from [60]. Copyright 2018, John Wiley and Sons. (e) Stretchable yarn electronic skin. Reproduced with permission from [61]. Copyright 2018, John Wiley and Sons. (f) Triple helical-structured cylindrical TENG. Reproduced with permission from [63]. Copyright 2019, American Chemical Society. (g) Amphibious yarn TENG. Reproduced under the terms of the Creative Commons Attribution 4.0 International License (CC BY-4.0) (https://creativecommons.org/licenses/by/4.0/) [62]. Copyright 2019, The Authors, published by Springer Nature.

Yarn TENG works based on the contact-separation of two or more fiber components. The first yarn TENG was achieved based on cotton threads by Zhou et al. in 2014 [57]. Cotton threads were firstly treated by nitric acid to increase the hydrophilicity, then coated with CNT by dipping and drying process to achieve the conductive threads as one of the fiber electrodes of TENG, another conductive CNT/cotton thread was further coated by polytetrafluoroethylene (PTFE) to serve as the triboelectric fiber with internal electrode, followed by twisting two fibers to construct the yarn TENG (Figure 3(a)), which works via the friction between two fibers. The average output power density can achieve ~0.1 µW cm^−2^. The device can be incorporated into a power shirt to trigger a wireless body temperature sensor system and detect human motion. In addition, Zhou et al. further coiled this yarn TENG around a silicone fiber to form an active yarn-based strain sensor in 2015 [56]. Its operation is based on the contact-separation of yarn TENG upon stretching, showing sensitive and stable performance to detect the strain up to 25% by virtue of the helical device based on a highly stretchable silicone fiber. Owing to the surface potential decay of PTFE coated fiber that can last up to 72 h, the quantitative measurement of transfer charges is realizable, the device was able to detect finger motion states.

Besides the external mechanical stimuli that could trigger the yarn TENGs, some efforts were also devoted to explore other actuations. Peng et al. firstly reported a novel photoelectric conversion yarn using photomechanical actuation and triboelectric effect in 2016. As shown in Figure 3(b) [58], a 1D yarn-type photo-activated TENG (photoelectric conversion device) was composed of two functional devices, a fiber TENG and a photothermal fiber actuator. TENG was fabricated by a kind of aligned CNT fibers with a coating of PTFE to generate and store charges. Optothermal component was a bilayer strip of aligned CNT sheet/paraffin wax and polyimide (PI) for photomechanical actuation. Under the periodical irradiation of visible light, the yarn device was repeatedly bent and released to change the gap between strip actuator and yarn TENG, inducing the charges generation by triboelectric effect to produce electricity. 70 mV of voltage can be produced by a typical device with a length of 3 cm. This kind of photoelectric conversion yarn was flexible and foldable, exhibiting tunable output voltages and durability under repetitive deformations.

Although both mechanical and optothermal stimuli are accessible to trigger the yarn TENGs, low electric output remains a challenge due to the limited area of yarns. Hence, various functional nanomaterials were employed to decorate the yarns for improving triboelectric performance. Hong et al. developed a hierarchically nanostructured 1D conductive bundle yarn-based (CBY) TENG in 2017 (Figure 3(c)). Starting from a kind of CBY, 2D ZnO nanowalls (NWs) were firstly grown on the CBY by hydrothermal synthesis, followed by depositing aluminum or ZnO nanoparticles to further increase the surface area for improvement of triboelectric effect. These decorated yarns can be designed as the arrays for mechanical interaction with a conductive fabric embedded in PDMS. A typical array composed of 10 yarns generates outputs of 78.1 V and 9.7 µA cm^−2^, respectively, showing highly improved triboelectric performance than that of a single yarn device without decoration [59].

In addition to high triboelectric output, good mechanical property is also important for the practical applications of yarn TENGs in multiple biomechanical energy harvesting and sensing. Wang et al. addressed this issue by incorporating polymer materials with the functional yarns in 2018. The first work, a stretchable core-sheath yarn TENG was designed in virtue of silver-coated nylon yarn and silicone rubber elastomer (Figure 3(d)) based on two parts with spring-like spiral winding structures [60], the internal core column is a silicone rubber fiber with winding of a conductive nylon yarn (inner electrode), a commercial elastic silicone rubber tube (triboelectric layer) serves as the external sheath frame, whose surface was twisted with another conductive nylon yarn (outer electrode) and followed by encapsulating by another layer of silicone rubber. The yarn TENG can harvest energy from omnifarious external mechanical stimuli such as compressing, stretching, bending, and twisting. The maximum outputs under various compressing frequencies (1–5 Hz) achieved 19 V and 0.43 µA while 13.5 V and 0.1 µA can be attained at a tensile strain of 100%. Multiple applications include self-counting skipping rope, self-powered gesture-recognizing glove, and a real-time golf scoring system were realized based on this smart yarn. The second work, a stretchable conductive yarn network was embedded into a planar silicone rubber film, resulting in a skin-inspired TENG with stretchability, good sensitivity and high precision for pressure sensing (Figure 3(e)), enabling a self-powered sensor to monitor human physiological signals, such as arterial pulse and voice vibrations [61].

High mechanical strength is of great importance to guarantee the subsequent weaving process. In addition, under the same stimulus from external force, the twisting method is also important for the electricity output. Recently, Kim et al. demonstrated that a triple helical-structured cylindrical TENG composed of a core-shell yarn can deliver higher triboelectric outputs (169 V and 18.9 μA) than the individual core-shell yarn device under the same compression (Figure 3(f)), inspiring a new choice to modify the yarn structure for improving the performance of wearable TENGs [63].

Yarns can be used to construct multistructured TENGs with higher output performance and good mechanical properties. However, the aforementioned yarns are only designed for presenting the configuration and possibility on TENGs. Owing to the challenges in the production of a yarn TENG with continuous length, it is still difficult to realize scalable incorporation or weaving as textile-device for high power outputs. Based on considerations of mass production of yarn TENG, recently, Wang et al. manufactured a type of continuous and scalable yarns that are capable of harvesting energy from both water and mechanical motion [62]. The core-sheath yarns were fabricated through blow-molding silicone rubber tube over the surface of stainless steel yarns using a specialized spinning equipment (Figure 3(g)). This amphibious energy yarn can work under various liquids include nitrogen, cyclohexane, methylbenzene, diethyl ether, ethyl acetate, phenylcarbinol, alcohol, and water, the maximum output can be achieved in water (~13 V) to power a liquid crystal display (LCD). In addition, this triboelectric yarn can be further twisted with another commercial waterproof yarn into double-plied yarns for weaving into the textile, which can generate electricity of ~22.9 V and 12.5 µW m^−1^ by stretching and pressing, achieving tunable outputs by changing the stretching frequency and the woven patterns of textiles. This scalable technology promises continuous yarn TENGs for weaving into textile TENGs, serving well for the practical applications.

### 4.2. Self-powered hybrid device based on fibers/yarns

**Figure 4.**
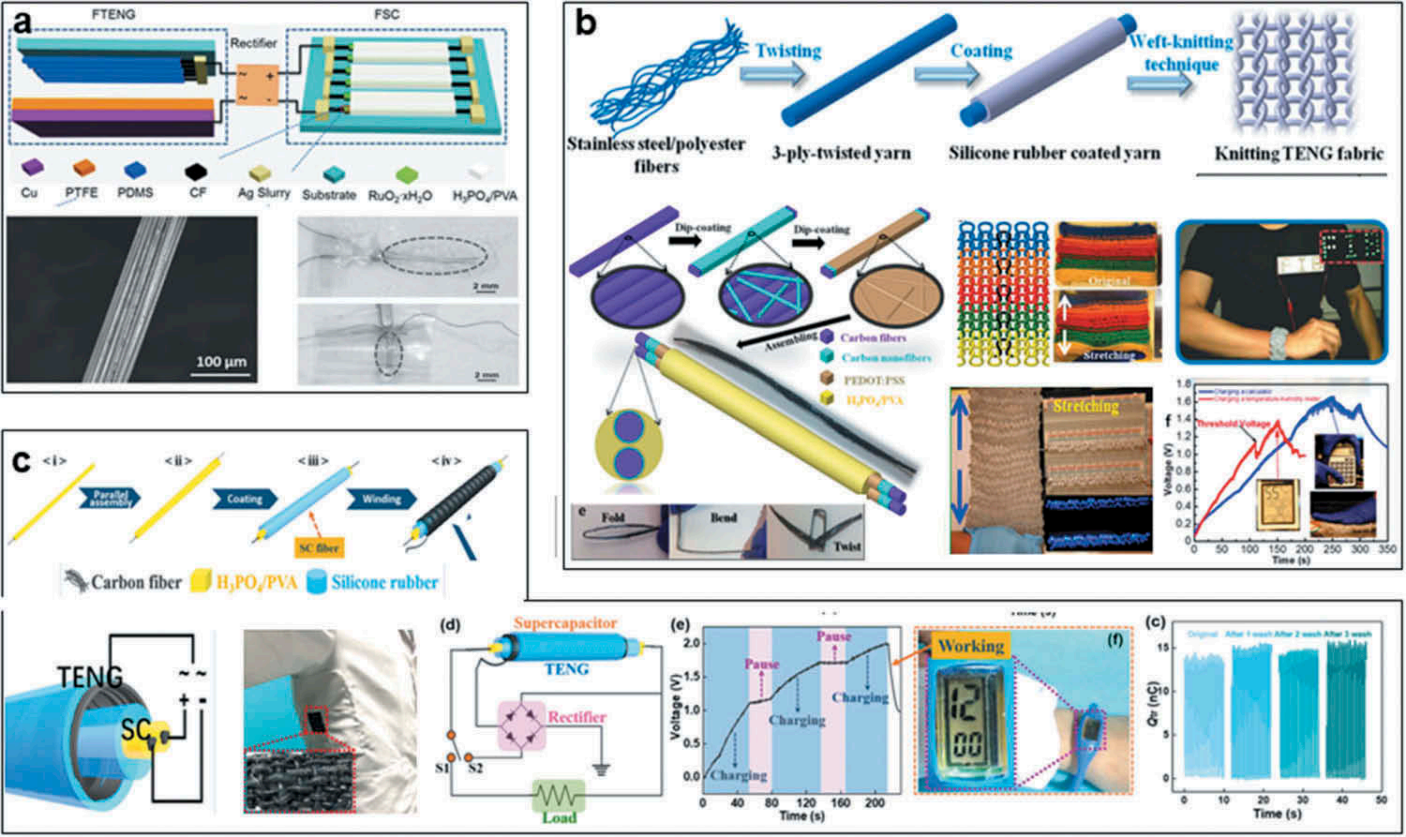
Hybrid devices based on fiber TENG. (a) Flexible fiber-based TENG-supercapacitor (SC). Reproduced with permission from [64]. Copyright 2015, John Wiley and Sons. (b) Stretchable and washable all-yarn integrated SC-TENG textile. Reproduced with permission from [65]. Copyright 2017, American Chemical Society. (c) Coaxial SC-TENG fiber-based self-charging fabric. Reproduced with permission from [66]. Copyright 2018, American Chemical Society.

Electricity can be generated by TENGs of fibers and yarns via harvesting the mechanical, optothermal, and water energy. However, to realize the wearable applications, storage of the output power is desirable for convenient usage. Therefore, efforts have been devoted to incorporate supercapacitor into 1D TENGs for both energy harvesting and storage. Wang et al. firstly set foot in this attempt in 2015, they designed a flexible and weavable 1D self-charging power system to harvest mechanical energy from human motions, the device consists of a fiber shape supercapacitor (FSC) and a fiber-based TENG (Figure 4(a)) [64]. RuO_2_·xH_2_O was synthesized on the surface of carbon fiber as the electrode of FSC, vapor-phase hydrothermal method renders the electrodes with high conductivity in both ion and electron, achieving FSC with a remarkable specific capacitance of 83.5 F cm^−3^ (3.2 mF cm^−1^ and 146 mF cm^−2^). Fiber TENG was designed based on PDMS coated carbon wire and a counter triboelectric material of PTFE. Two devices were assembled into an effective self-charging power system as separate mode (Figure 4(a)).

To improve the practicability, a highly stretchable and washable all-yarn-based self-charging knitting power textile was further realized by Wang et al. in 2017. SC and TENG were fabricated separately as illustrated in Figure 4(b), followed by weaving into a self-powered textile to harvest and store biomechanical energy [65]. It can generate a maximum instantaneous power density of ~85 mW m^−2^ and light up approximately 124 light-emitting diodes (LEDs), promising the power sources for wearable electronics such as calculator and temperature-humidity meter.

However, hybrid systems consisted of separate configurations, because SC and TENG were fabricated on different fibers. To achieve dual functions in individual fibers/yarns, Sun et al. developed a coaxial fiber with a supercapacitor inside and a TENG outside in 2018 (Figure 4(c)), which can not only harvest mechanical energy but also store energy in the all-in-one fiber [66]. Carbon fiber yarns were employed as electrode materials for both TENG and SC. Meanwhile, silicone rubber serves as triboelectric material for TENG to separate with SC, it was also used as encapsulation for the whole fiber device, which could be further knitted as self-charging power fabric for sustainably power wearable electronic devices. This work indicates the incorporation feasibility of TENG and SC as the 1D fiber/yarn devices, promising platform for wearable electronics and smart textiles.

### 4.3. Potential and challenges of yarn TENGs

Compared to coaxial fiber TENGs, yarn TENGs are more processable with richer configurations. This is because more fibers can be contained within the devices, providing higher outputs as well as better mechanical properties for subsequent incorporation and weaving. Besides, most of the yarn TENGs can be fabricated as a demo to present the feasibility of those ideas and configurations, a continuous and scalable yarn TENG has been achieved, and additional waterproof performance has been addressed to extend the application potential (Figure 3(e)). The sole concern on this kind of yarn TENG would be the stainless steel fiber electrode, which would decrease the conformability, washability, and wearability of the resultant electronic textiles. Therefore, improved continuous 1D electrode would be the next direction of efforts for the mass production of yarn TENGs.

## 5. Textile-based TENGs

Continuous triboelectric and electrode materials with desired processability and wearability would be a challenge in 1D processing. This issue potentially can be addressed with 2D plane substrates such as based on fabric or textile, which could provide more choices on materials and configurations for the fabrication of TENGs. Textile, a planar pattern woven by fibers or yarns for processing into functional fabric or garment, is also the desirable ultimate form of substrates for wearable electronics. Tremendous efforts have been made to fabricate various TENGs based on fabrics or textiles, including improving the triboelectric performance, operability, and washability of devices. In general, there are two routes to achieve the textile TENGs, the first one is fabricating the TENGs based on yarns, threads, and belts, followed by weaving those units into textile devices to improve the applicability [67–85]. The second way is realizing TENGs by *in* *situ* functionalization directly on the off-the-shelf textiles that were woven by synthetic or natural fibers/yarns [86–100]. In the following section, we summarize the progress on textile TENGs that were realized by the two routes and compare their advantages and disadvantages in materials, processing, and performances.

### 5.1. Textile TENGs by post weaving

**Figure 5.**
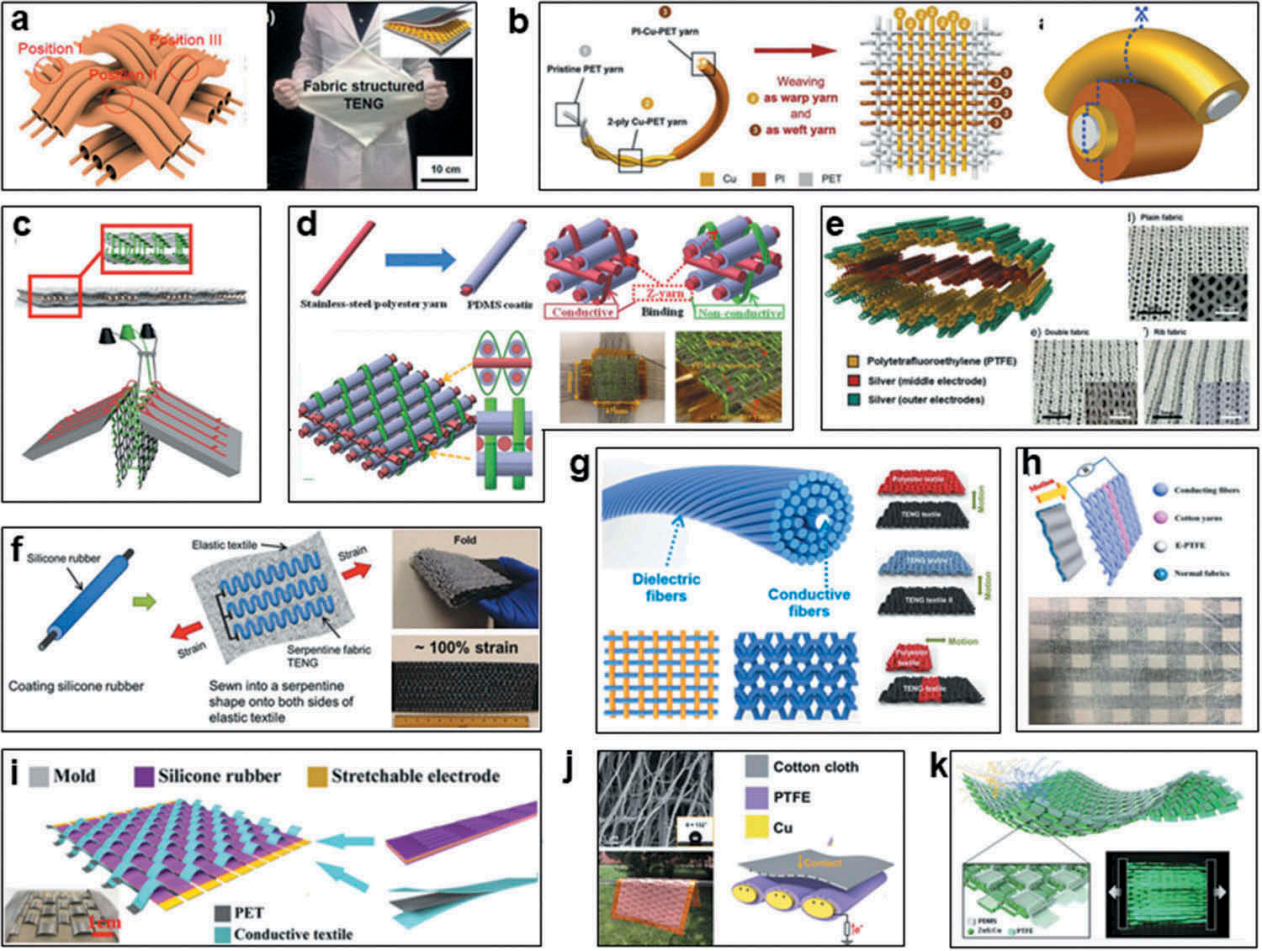
Textile TENGs by post integrating/weaving. (a) Stretchable 2D fabric TENGs based on Al fibers. Reproduced with permission from [69]. Copyright 2015, American Chemical Society. (b) Washable textile TENG with PET fibers. Reproduced with permission from [71]. Copyright 2016, John Wiley and Sons. (c) Nylon textile TENG with 3D fabric spacer. Reproduced with permission from [68]. Copyright 2016, Elsevier. (d) 3D orthogonal woven silicone/polyester textile TENG. Reproduced with permission from [72]. Copyright 2017, John Wiley and Sons. (e) Stretchable knitted PTFE textile TENGs. Reproduced with permission from [73]. Copyright 2017, American Chemical Society. (f) Sewable silicone textile TENG. Reproduced with permission from [74]. Copyright 2017, John Wiley and Sons. (g) Textile TENG with conductive core-shell yarns. Reproduced with permission from [77]. Copyright 2017, American Chemical Society. (h) Knitted-patterned cotton-based TENG. Reproduced with permission from [84]. Copyright 2019, Elsevier. (i) Tailorable textile TENG with PET/silicone belts. Reproduced with permission from [78]. Copyright 2018, American Chemical Society. (j) Washable porous PTFE/Cu-based textile TENG. Reproduced with permission from [82]. Copyright 2018, Royal Society of Chemistry. (k) Self-powered motion-driven triboelectric electroluminescence textile. Reproduced with permission from [85]. Copyright 2019, American Chemical Society.

Wang et al. firstly realized the textile TENG in 2014 by readily incorporating nylon fabric, polyester fabric, and silver fiber fabric. Based on the friction between different fabrics, the device can be operated under vertical contact-separation mode and lateral sliding mode, and can be incorporated in clothes to harvest mechanical energy from human body [67]. Thereafter, Baik et al. achieved a stretchable and waterproof 2D fabric TENG for application under harsh environment in 2015 (Figure 5(a)) [69]. The device was fabricated based on the separate design of coaxial fiber TENGs, fiber device was composed of an Al wire core and a PDMS tube as the shell. ZnO nanowires were first grown on the Al wire by hydrothermal, followed by depositing an Au thin film to produce a 3D Au/Al branched fiber. PDMS was etched by RIE (reactive ions etching) to produce the nanowire arrays inside the tube. Then, Au/Al fiber was inserted into the PDMS tube with additional encapsulation of Al foil to achieve the fiber TENG, followed by weaving to attain the fabric TENG with high outputs of 40 V and 210 µA, it could serve as a power mat during walking and power clothing attached to the elbow.

Thereafter, to achieve the scalable fabrication of textile TENG, Zheng et al. directly woven Cu-coated polyethylene terephthalate (Cu-PET) warp yarns and polyimide (PI)-coated Cu-PET weft yarns on an industrial sample weaving loom (SL8900S, CCI) in 2016, resulting in a washable textile with sensitive deformation and effective triboelectricity generation even upon gentle tapping or bending, a maximum short-circuit current density of 15.5 mA m^−2^ can be achieved (Figure 5(b)) [71]. Superior sensitivity makes it can be integrated into a chest strap to monitor human respiratory information of rate and depth.

Woven structure of textile is diverse, providing an effective way to realize the novel design for improving the triboelectric performance of textile TENG. Zhi et al. designed a 3D knitted fabric as spacer, combining with two nylon fabrics with coating of graphene (electrode) and PTFE (triboelectric layer), respectively, producing an all-fabric TENG operated based on vertical contact electrification, which can generate outputs of 3 V and 0.3 µA (Figure 5(c)) [68]. This kind of knitted fabric spacer solves the compatibility of wearing and mechanized production of textile TENGs.

Wang et al. achieved a kind of 3D orthogonal woven TENG in 2017 by combining the stainless steel/polyester fiber-blended yarns with and without a coating of PDMS, and nonconductive binding yarn as shown in Figure 5(d). 3D structural design renders the maximum power density of 263.36 mW m^−2^ can be achieved on the textile under tapping of 3 Hz [72]. It can serve as a self-powered motion sensor to monitor the movement signals of human body, as well as a dancing blanket to harvest biomechanical energy and detect body movement.

Furthermore, by using knitted fabrics, Kim et al. achieved a fully stretchable textile TENG in 2017 (Figure 5(e)), the device was adaptable to cloth movement and was able to generate electricity under compression and stretching [73]. Investigation of plain fabric, double fabric, and rib fabric indicates the rib-knitted fabric shows superior stretchability, enabling dramatic increasing in triboelectric outputs upon stretching due to the increased contact surface. The maximum outputs reach up 23.5 V and 1.05 µA under strain of 30%, 60 µW can be attained under compression at 3.3 Hz.

Therefore, woven structure that is useful for regulating warming property and mechanical performance for traditional textiles is also of great importance for improving the triboelectric outputs due to their varying surface roughness. Besides, a kind of sewable thread TENG was demonstrated by Wang et al. in 2017 [74], the device was composed of one silicone-rubber-coated thread, which was sewed into a serpentine shape on an elastic textile, achieving a highly stretchable and scalable textile TENG for scavenging human-motion energy (Figure 5(f)). Wireless wearable keyboards and smart beds were proved to indicate its functions for gesture sensing, human-interactive interfaces, and physiological signal monitoring. Similarly, a kind of scalable core-shell yarns was further demonstrated by Wang et al. based on a conductive fiber with wrapping of polymer/natural fibers bundle (Figure 5(g)) [77], the continuous yarns can be woven/knitted into energy textiles that are comfortable, fashionable, washable and tailorable. It can work under various modes including single-electrode mode, contact-separation mode as well as free-standing mode, promising for further garment processing. Furthermore, Zhu et al. designed a patterned textile by knitting the conductive silver-plated nylon threads and cotton yarns, followed by laminating with a composite fabric to realize the free-standing mode textile TENG (Figure 5(h)) [84], opening a new way to explore large-scale triboelectric textiles with improved outputs for wearable power sources.

Stretchable textile TENG not only depends on the woven structures, but it is also realizable by some special design on the units. In addition to the conductive fibers/threads such as silver fiber, stainless steel, and metal coated-polymer fibers can serve as the electrodes, a specially designed stretchable belt electrode has been proved for textile TENG by Chou et al. in 2018 [78]. Silicone rubber (TN-920) mixed with silver-coated glass microspheres as a stretchable electrode, which was encapsulated by another silicone layer, followed by patterning by hot-press and reverse mold technology to result in the stretchable patterned belts (Figure 5(i)). They were integrated with Ni-coated PET fabrics to construct the stretchable TENG, it can generate an instantaneous open-circuit voltage of 350 V and power of 1 mW based on friction between the knitted units, 32 V can be achieved upon stretching. Coupling with a cotton fabric, improved output can be attained. The stretchable textile TENG can harvest energy from multivariant human body motions, providing a new approach to realize stretchable textile electrodes [101–103] for TENGs.

Moreover, the triboelectric performance can be further improved by exploring new fibers/yarns. Mao et al. designed a washable single-electrode triboelectric textile for biomechanical energy harvesting in 2018 (Figure 5(j)) [82]. It was woven by the copper foil strips that were prewrapped by a kind of specially designed porous PTFE strips, which were fabricated using PTFE fine powder and porogenic agent of naphtha (25%) via thermal extrusion. This kind of porous PTFE strips not only endows the textile with resistance to stains produced by various agents such as milk, oil, ink, cola, juice, honey, jam, and sands, maintaining stable triboelectric outputs after washing them away, but also highly enhance the ability in converting the mechanical energy, a textile device woven in clothes can generate outputs of 1050 V and 22 µA upon swinging arms, the power was enough to drive a night running light and a digital watch, showing an effective approach to produce textile with enhanced triboelectric performance.

These self-powered textiles have been widely presented to serve as the power sources and human motion sensors. More interesting, a novel integration of self-powered motion-driven triboelectric electroluminescence (EL) textile has been demonstrated by Kim et al. recently (Figure 5(k)). The textile was realized by weaving the belts of ZnS:Cu/PDMS composite and PTFE fibers [85]. Mechanical stimuli could induce deformation of the textile to generate tribo-electrification, the instantaneous triboelectric filed could be produced from the contact-separation between PDMS and ZnS:Cu microspheres, as well as the contact objects and textile surface. Therefore, EL belts could be lighted twice in a contact cycle, enabling continuous light could be emitted by various movements. This work extends the application potential of triboelectric effect, demonstrating a new route to develop the self-powered systems for wearable electronics.

### 5.2. Textile TENGs fabricated by in situ functional finishing

Using the abovementioned methods of post weaving, precise designs are addressed in each unit of fiber and yarn, important progress has been achieved in developing the textile TENGs by optimizing fibers/yarns-based devices in terms of materials, morphologies, and woven structures. However, most of the reported works are mainly incorporating various polymers materials and wires into the 1D TENGs to configure the triboelectric textiles. Although the triboelectric performance and durability of devices are guaranteed, limited efforts have been made to cover the breathability, comfort, and wearability of textile TENGs. These concerns could be addressed by the second route of *in* *situ* functional finishing. A lot of work has been carried out by coating various triboelectric materials and conductive materials on the textiles, there is no limitation on the dimension of textile, promising the scalable process for self-powered wearable systems [86–100]. In the following section, we summarize the progress on triboelectric textiles fabricated by *in* *situ* functional finishing, such as various coating methods, deposition, reactive ion etching (RIE), and chemical modification, and we compare the advantages of different processes on the comprehensive properties of textile TENGs, including the triboelectric output, washability, durability, and comfort level as well as wearability.

**Figure 6.**
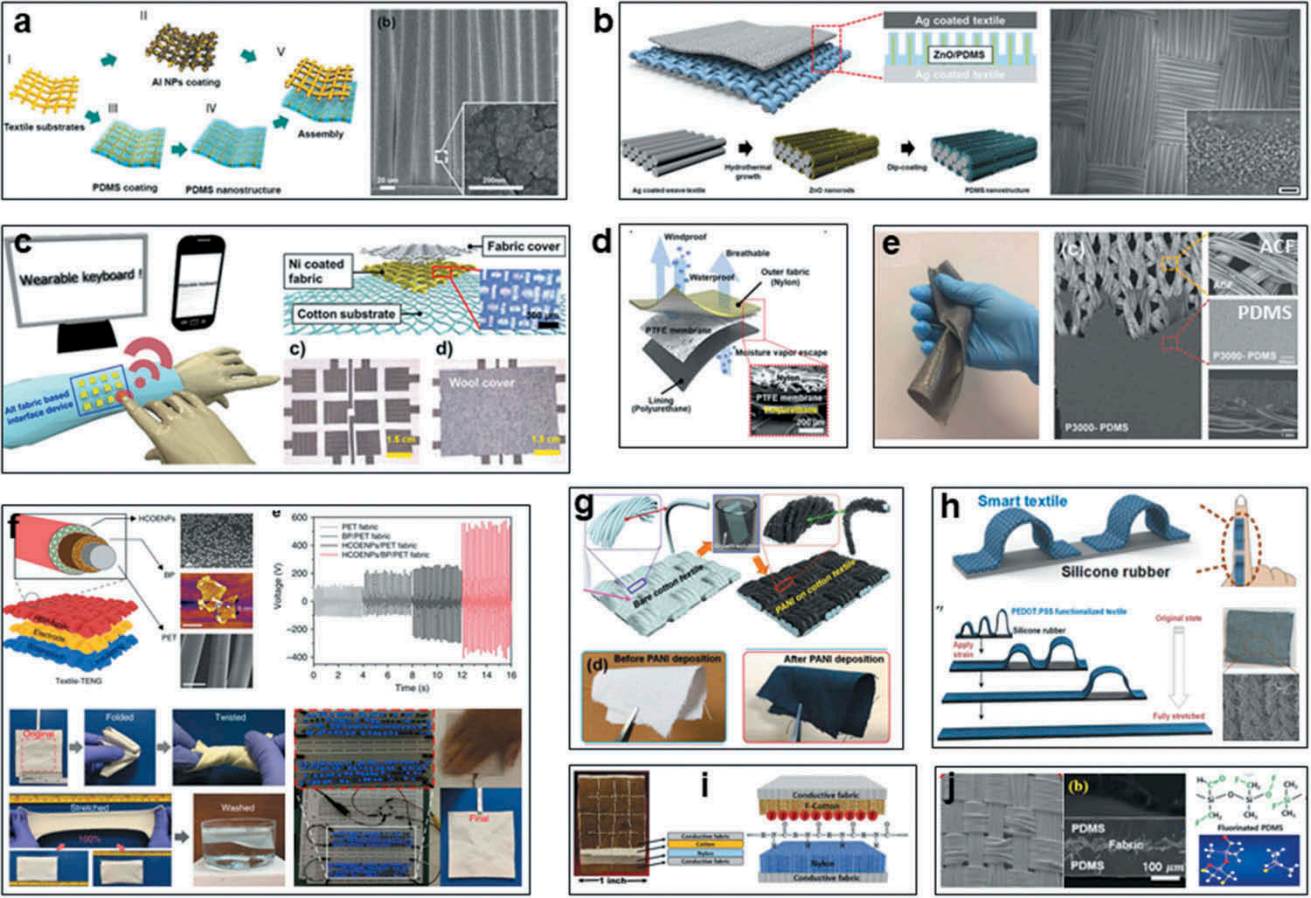
Textile TENG by *in* *situ* functional finishing. (a) PDMS nanostructured textile TENG. Reproduced with permission from [87]. Copyright 2015, Elsevier. (b) Ag/ZnO/PDMS nanopatterned textile TENG. Reproduced with permission from [88]. Copyright 2015, American Chemical Society. (c) Cotton-based self-powered keyboard. Reproduced with permission from [92]. Copyright 2018, Elsevier. (d) Nanodot-patterned PU textile TENG. Reproduced with permission from [93]. Copyright 2018, Elsevier. (e) Chinlon textile TENG with PDMS/Ag coating replicated by sandpaper. Reproduced under the terms of the Creative Commons Attribution 4.0 International License (https://creativecommons.org/licenses/by/4.0/) [94]. Copyright 2018, The Authors, published by MDPI. (f) Skin-actuated PET textile TENG. Reproduced under the terms of the Creative Commons Attribution 4.0 International License (https://creativecommons.org/licenses/by/4.0/) [86]. Copyright 2018, The Authors, published by Springer Nature. (g) PANI-coated cotton textile TENG. Reproduced with permission from [97]. Copyright 2019, Elsevier. (h) Multi-arch strain sensor enabled by PEDOT:PSS coated textile. Reproduced with permission from [98]. Copyright 2019, Elsevier. (i) Fluoroalkylated polymeric siloxanes functionalized cotton TENG. Reproduced with permission from [89]. Copyright 2019, John Wiley and Sons. (j) RIE surface-engineered PDMS fabric TENG. Reproduced with permission from [100]. Copyright 2019, Elsevier.

Hong et al. developed a textile TENG based on a kind of commercial Au-coated textile in 2015 (Figure 6(a)), which serves as electrodes, and coated with nanostructured surface by Al nanoparticles and RIE-PDMS. Power density of 33.6 mW cm^−2^ can be achieved on the device upon bending, and the device can be attached on clothes to harvest energy from human arm to power LED [87]. However, the breathability of the device is a concern due to one of the textiles was fully covered by PDMS, rendering poor affinity and low comfort. Thereafter, an improved textile TENG was fabricated by Kim et al. (Figure 6(b)), a commercial Ag-coated textile serves as the electrodes, one of them was grown with ZnO nanorods by hydrothermal process, followed by coating with a thin layer of PDMS for the triboelectric layer [88]. The device is operated based on the interaction between Ag-coated textile and PDMS, high outputs of 120 V and 65 µA can be achieved, improved outputs of 170 V and 120 µA are attained from a four-layer-stacked TENG, which can power LEDs, LCD, and a keyless vehicle entry system.

To further improve the conformability and applicability of textile TENG, Jin et al. designed a self-powered wearable keyboard fabric using triboelectric effect in 2018 (Figure 6(c)), the device possesses a sandwich structure that comprised of a cotton substrate, Ni-coated fabric, and a wool fabric cover [92]. Twelve square devices with a length of 1.5 cm as cells were patterned into a keyboard, which can detect a keystroke precisely, and capable of typing a word and play music. Besides using hand, multiple choices of the counter materials were conformed can be polyester, cotton, and wool. The whole device was foldable, durable and washable, proposing a simple way to utilize triboelectric effect for actual applications.

Plain coating is not enough to achieve the desired power density due to the limited surface roughness. Therefore, Hong et al. realized a kind of gold nanodot-pattern by electron-beam sputtering and oxygen plasma etching on polyurethane textile to enhance the triboelectric effect in 2018 (Figure 6(d)) [93], it was assembled with another Au-coated nylon fabric and a spacer of PTFE membrane, constructing a breathable and washable textile TENG, which works via lateral sliding mode for mechanical energy harvesting. It can be used as a flutter membrane between two Al electrodes for wind energy harvesting, showing a maximum power of 70 µW. To reduce the processing cost, a more efficient method for rough surface was subsequently addressed by Li et al., Ag-coated chinlon fabric (ACF) was coated by PDMS, surface roughness was able to be replicated from sandpaper to effectively enhance the triboelectric outputs (80.4 V) (Figure 6(e)) [94].

Although considerable outputs and effective applications have been achieved by incorporating functional polymers and conductive materials on the textiles, these devices did not fully exhibit the advantages of textile for wearable electronics due to the porous structure and breathability of textile have been screened. Comfortability and wearability of textile TENGs are still unattainable. Therefore, by using functional nanocoating, Lee et al. designed a kind of skin-actuated sandwich-structured textile TENG (single electrode mode) with conformability, comfortability, and washability for durable biomechanical energy harvesting in 2018 (Figure 6(f)) [86]. A fabric electrode with silver flakes and PDMS binder was employed to achieve the stretchability and breathability of the device. A waterproof textile with a transparent coating of cellulose-derived hydrophobic nanoparticles (HCOENPs) was applied to encapsulate the whole device for the water repellency and washability [104]. HCOENPs have no effect on the color of textile, showing the potential of cellulose materials for flexible electronics [105]. The triboelectric textile layer was realized by virtue of a synergetic triboelectric trapping layer of black phosphorus (BP) with protection by HCOENPs, ensuring the favorable comfortability and wearability. Of which, both BP and HCOENPs can effectively enhance the triboelectric effect, HCOENPs encapsulation layer could provide additional protection for BP to alleviate the degradation. BP layer under HCOENPs could suppress the charge loss to ensure a more complete transfer of induced charges, rendering the enhanced output of ~250–880 V and ~0.48–1.1 µA cm^−2^ under gentle touch with small force (~5 N) and low frequency (~4 Hz), as well as sensitive capability to harvest energy from both voluntary and involuntary body motions. It is capable of driving more than 150 LEDs and a digital watch. This all-textile TENG delivers long-term reliability and high triboelectricity regardless of various extreme deformations, severe washing, and extended environmental exposure, promising excellent wearability for self-powered systems.

Thereafter, some other coating processes with conductive polymers have been reported to fabricate the textile TENGs with good breathability and wearability. Yu et al. designed a cotton textile-based TENG by coating with polyaniline (PANI) via low-temperature *in* *situ* polymerization in 2019 (Figure 6(g)) [97]. The PANI-cotton textile serves as both positive triboelectric material and electrode to construct TENGs in single-electrode mode and dual-electrode mode. The devices are highly incorporable with clothes for harvesting biomechanical energy and driving portable electronics. The maximum outputs reach up to 350 V and ~45 µA. Similarly, PEDOT:PSS was dip-coated on textile by Lee et al. to fabricate TENG by combining with a PTFE film (Figure 6(h)) [98]. A height-varying multi-arch strain sensor with large strain sensing range from 10% to 160% was developed, it can be mounted on human fingers to monitor hand gestures, also can be located at different body locations to track human activities, as well as using for a wearable CO_2_ sensor. It is promising to be incorporated in clothes as multi-functional self-powered sensors.

Physical coating processes with suitable materials demonstrate the facile and effective means to endow textiles with desirable triboelectric performance, and maintain the merits of textiles. In addition, chemical modification is also a feasible way especially for improving the triboelectric output of textile by increasing the surface charge density. Andrew et al. realized a functionalized cotton fabric by incorporating fluoroalkylated polymeric siloxanes in 2016 (Figure 6(i)), making the cotton fabric ranking at a more negative location in triboelectric series [89]. Kelvin probe force microscopy confirmed the increased surface charge density of the resultant cotton, the outputs can be increased to 4 times compared to the pristine cotton. Recently, Hong et al. coating PDMS on the Ni-Cu fabric firstly, followed by treating by air-plasma and RIE treatment to obtain a fluorinated PDMS (Figure 6(j)) [100]. Owing to the large electronegativity of fluorine (F), enhanced triboelectric output can be yielded on the F-textile by the aid of the efficient surface charging ability.

### 5.3. Textile TENGs for water energy harvesting and sensing

**Figure 7.**
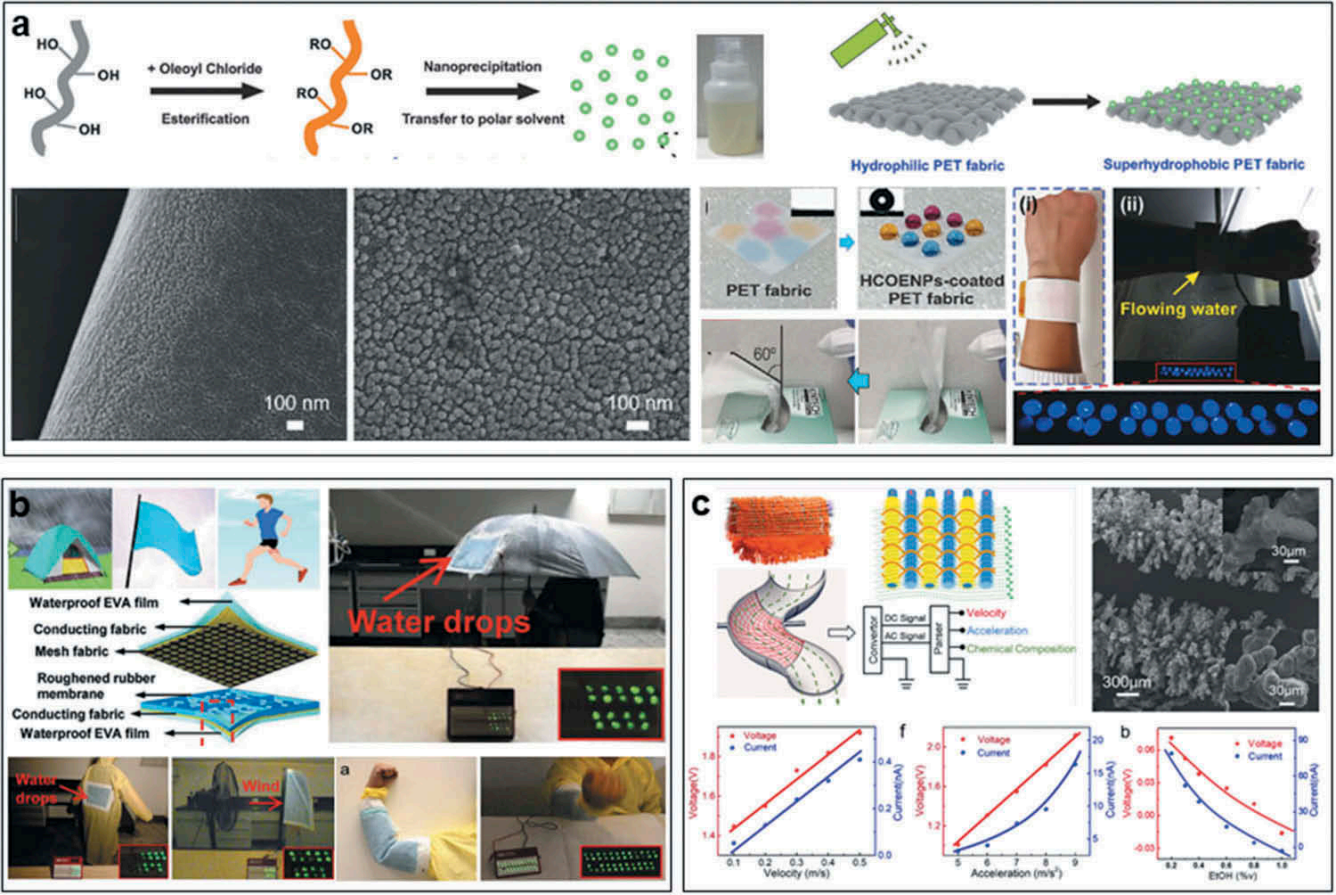
Coating-enabled textile TENG for water energy harvesting and sensing. (a) Cellulose-derived breathable and wearable all-fabric water TENG. Reproduced with permission from [91]. Copyright 2017, John Wiley and Sons. (b) Roughened rubber/EVA-based fabric TENG. Reproduced under the terms of the Creative Commons Attribution 4.0 International License (CC BY-4.0) (https://creativecommons.org/licenses/by/4.0/) [99]. Copyright 2019, The Authors, published by John Wiley and Sons. (c) PTFE/micrometal dendrites textile for all-immersed fluid sensor. Reproduced with permission from [95]. Copyright 2018, American Chemical Society.

In addition to the desired mechanical energy at dry conditions can be harvested in virtue of functional coatings, which would also contribute additional properties such as water resistance for textiles. It is significant to extend the applications of those textiles, such as water energy harvesting and fluid sensing [106–110]. Some works have been explored in this field, opening a new application of textile for self-powered wearable system in all-weather conditions. In 2017, Lee et al. realized energy harvesting from water flow based on our daily textiles for the first time (Figure 7(a)) [91]. A kind of cellulose-derived nano-coating was designed to readily achieve the waterproof textile with self-cleaning ability, air-permeability, and anti-fouling property. The textile was designed into single-electrode TENG for harvesting electrostatic energy from water. A dual-mode TENG consisted of a single-electrode device and a dual-electrodes device was realized, which can harvest both electrostatic energy and mechanical energy from water flow. The optimized textile TENG with a size of 3 by 3 cm can generate outputs of 22 V and 8 µA, lighting up more than 20 LEDs. Owing to the largely maintained merits of textile, this device can be directly sewed into glove as well as be designed as a wristband to harvest water energy, showing excellent breathability, comfortability, and wearability for self-powered applications under water conditions.

Thereafter, Lai et al. designed a waterproof textile TENG based on conductive fabric with combination of a roughened silicone rubber and an encapsulation layer of EVA film, resulting in an adaptive, wearable, and universal energy collector, which can harvest energy from various stimuli such as rain, wind, and multiple body movements (Figure 7(b)). It can be incorporated into the garment for remotely controlling a music-player system, promising the democratic collections of alternative energy for the self-powered wearable systems [99].

Besides the energy harvesting from water flows and droplets, waterproof textiles with triboelectric effect could serve as self-powered sensor in a fully immersed condition. Recently, Fan et al. reported a flexible self-powered all-in-one fluid sensor textile using interfacial triboelectric effect of arrays of a kind of micrometal dendrites (Figure 7(c)) [95]. PTFE nanofibers were coated on the dendritic cable electrodes to further enhance the triboelectric response. When completely immersed in the fluid, the textile can monitor the velocity, acceleration, and chemical composition by the aid of the regular electric signals, proposing a potential self-powered fluid sensor for precise industry, monitoring leakage or blockage inside chemical and petroleum pipelines. These few works indicate triboelectric textiles enabled by functional coatings are promising to be applied in various extreme conditions, serving as wearable power source and self-powered sensor.

### 5.4. Self-powered textile hybrid devices

**Figure 8.**
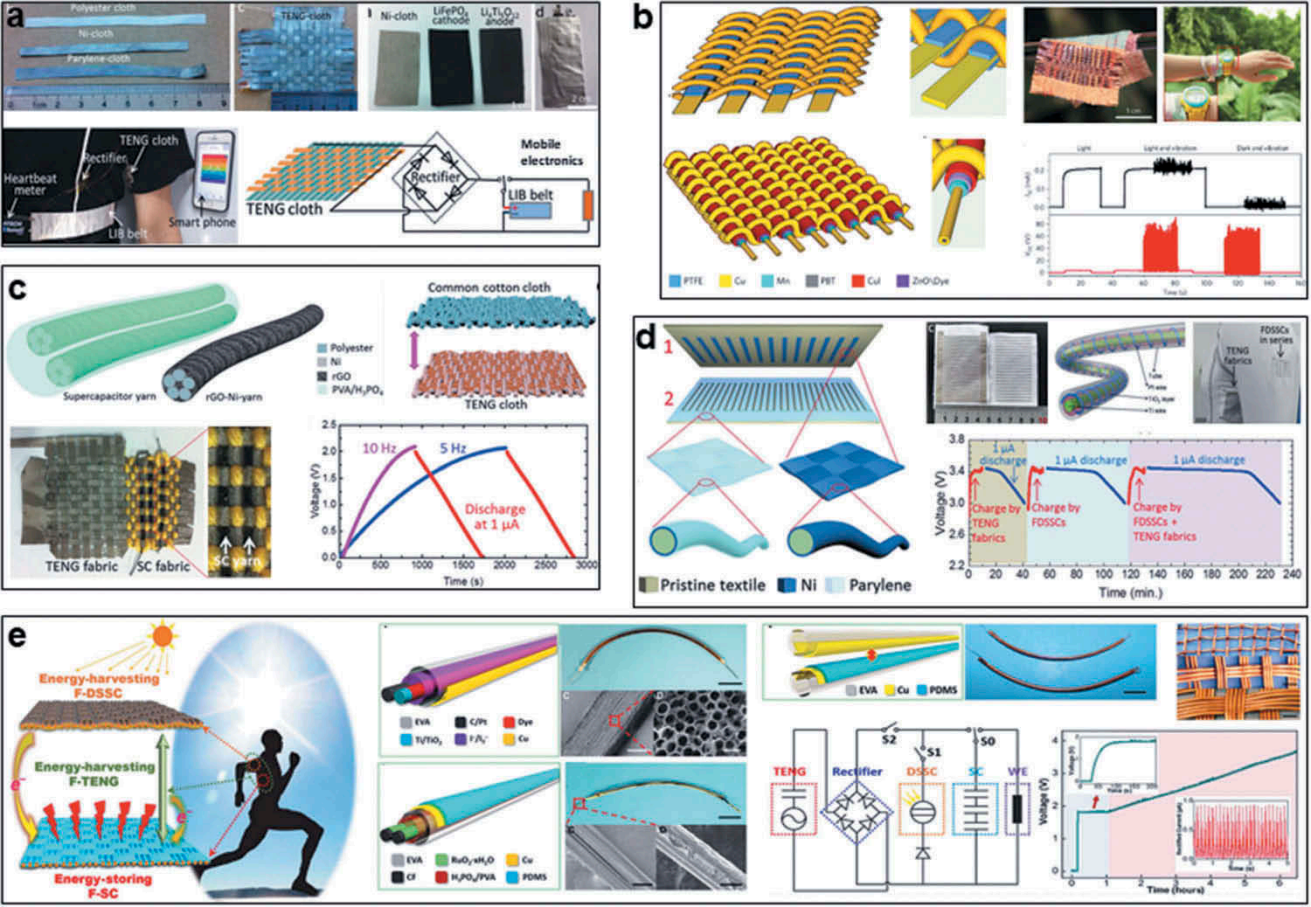
Hybrid devices based on textile TENG. (a) Power textile integrated by TENG and lithium-ion-battery (LIB). Reproduced with permission from [113]. Copyright 2015, John Wiley and Sons. (b) Micro-cable structured textile integrated by TENG and solar cell. Reproduced with permission from [114]. Copyright 2016, Springer Nature. (c) Self-charging textile integrated by fabric TENG and yarn supercapacitor (SC). Reproduced with permission from [115]. Copyright 2016, John Wiley and Sons. (d) Power-textile integrated by fabric TENG and fiber dye-sensitized solar cells (FDSSCs). Reproduced with permission from [116]. Copyright 2016, John Wiley and Sons. (e) Self-powered textile hybridized by fiber-shaped TENGs, FDSSCs, and SCs. Reproduced under the terms of the Creative Commons Attribution 4.0 International License (CC BY-4.0) (https://creativecommons.org/licenses/by/4.0/) [117]. Copyright 2016, The Authors, published by the American Association for the Advancement of Science.

Different triboelectric outputs can be attained by changing the materials choice and structure design of textile TENGs, integration of energy harvester and storage device is important to realize the application of electricity [111,112]. TENGs can be integrated with solar cell, battery, and supercapacitor, achieving multiple energy harvesting, and storage. Wang et al. firstly realized the combination of TENG-cloth and flexible lithium-ion battery (LIB) in 2015 (Figure 8(a)) [113]. Starting from a polyester belt, coating with Ni film by electroless plating to serve as both electrodes for TENG-cloth and as current collectors for the LIB belt. Parylene was coated on another Ni-belt to be applied as triboelectric units. TENG-cloth can convert the mechanical energy of human motions into electricity, it was proved to charge the LIB for three cycles, utilization efficiency of 72.4% power can be achieved between TENG-cloth and LIB. The charged LIB can power a heartbeat meter strap that is able to remotely communicate with a smartphone, presenting the feasibility of a self-charging wearable system enabled by triboelectric device.

Thereafter, Wang et al. further developed a micro-cable structured textile in 2016 for harvesting both solar and mechanical energy (Figure 8(b)) [114]. Solar cells were fabricated into micro cables based on the lightweight polymer fibers, followed by weaving with fiber-based TENGs via shuttle-flying process to create a smart fabric with a thickness of 320 µm. TENG was composed of PTFE stripes and copper-coated polymer fibers. Copper electrodes also serve for photovoltaic textile, the lightweight polymer fiber was employed as a substrate of photoanode to simplify the configuration. By harvesting energy under ambient sunlight and mechanical excitation, a textile with a size of 4 cm by 5 cm was able to charge a 2 mF capacitor to 2 V in 1 min. It was demonstrated to continuously power a digital watch, charge a cell phone and drive water splitting reactions.

Furthermore, Wang et al. realized the integration of an all-solid-state yarn supercapacitor (SC) and fabric TENG in 2016 to obtain a wearable self-charging power textile (Figure 8(c)) [115]. Ni layer and reduced graphene oxide film were successively coated on the surface of polyester yarns, with use of poly (vinyl alcohol) (PVA)/H_3_PO_4_ gel as solid electrolyte and separator, resulting in a bendable (180°, 1000 cycles) symmetric yarn SC with both high capacitance (13 mF cm^−1^, 72. 1 mF cm^−2^) and stable cycling performance (96%, 10,000 cycles). On the other hand, fabric TENG was fabricated by weaving using Ni-coated polyester straps and parylene-Ni-coated polyester straps. It can generate outputs of 40 V and 5 µA upon motion at 5 Hz. Therefore, yarn SCs and fabric TENG can be woven and integrated into an individual textile. Three-series yarn SCs were proved to be charged by TENG to 2.1 V in 2009 s under impacting of 5 Hz, charging time can be shortened to 913 s while using stimulus of 10 Hz. The charged SCs can be discharged at 1 µA for 811 s, exhibiting application potential of this self-charging power for wearable systems.

In addition, fiber-shaped dye-sensitized solar cells (FDSSCs) also can be integrated with textile TENG, which was realized by Wang et al. in 2016 (Figure 8(d)) [116]. They designed a grating-structured fabric TENG via a route of laser-scribing masking and electroless deposition Ni plating, obtaining the conductive circuits and parylene pattern on the textiles, resulting in textile TENG that can be operated under lateral sliding mode with tunable outputs by changing the sliding speed, the optimized outputs reach up 120 V and 55 µA at 0.75 m/s. 1D fiber FDSSCs were fabricated based on a Ti wire, TiO_2_ layer and Pt wire were successively coated on Ti wire to serve as the separator and the counter electrode, followed by sealing via a transparent PTFE. The FDSSCs can be woven or sewed into the textile TENG to realize a self-charging system.

More interesting, a self-powered textile composed of hybrid fiber-shaped TENG, solar cells (FDSSCs), and supercapacitors (FSCs) was further realized by Wang in 2016 (Figure 8(e)). It can simultaneously harvest energy from sunshine and body motions, and store them in the SCs for wearable applications [117]. FDSSCs were fabricated starting from a working electrode of Ti wire that was coated by TiO_2_ nanotube arrays, Pt-coated carbon fiber serves as the counter electrode, followed by sealing via Cu-coated EVA tubing with electrolyte, attaining a bendable FDSSC (diameter ~10 µm) with a stable current density of ~12 mA cm^−2^. Conductive EVA tube serves as both holder for FDSSC and one electrode for TENG. FSCs were fabricated based on two carbon yarns that with a coating of RuO_2_·xH_2_O in H_3_PO_4_/PVA electrolyte, followed by packing with a PDMS/Cu/EVA tube, which was used for both holder of FSC and another electrode of TENG. The resultant FSCs is bendable and shows stable specific capacitance of 1.9 mF cm^−1^ and energy density of 1.37 mJ cm^−1^. Therefore, a TENG can be assembled by one FDSSCs fabric and one FSCs fabric (Figure 8(e)), generating the maximum outputs of ~12.6 V and 0.91 µA. Furthermore, a hybridized self-charging power textile was realized by integrating the above three devices, FDSSCs fabric and TENG can work together to harvest the solar energy and mechanical energy, respectively, charging the FSCs fabric. This work demonstrates the possibility to meet the increasing demand for lightweight, stretchable, washable, and wearable power source for self-charging electronic textiles.

### 5.5. Potential and challenges of textile TENGs

In short, both post weaving and *in* *situ* functional finishing can be used to fabricate textile TENGs. Firstly, post weaving based on the fibers/yarns TENGs can realize the precise design of TENG in each unit, improved triboelectric outputs are achievable using various woven patterns and knitted structures such as plain fabric, satin fabric, and rib fabric. It renders easy integration of different functional devices, such as TENG, solar cell, supercapacitor, and battery, realizing the hybrid self-charging power textiles. Also, in this way, the woven textile devices could maintain the favorable air-permeability if the functional fibers/yarns are guaranteed to be flexible with small diameter and enough length, even it is a challenge currently. Accordingly, we should also take note that the sewing or weaving highly depends on the mechanical properties of fiber/yarn devices, and the stability of their electric performances needs to be considered during the subsequent processing and operation.

In comparison, *in* *situ* functional finishing could provide more material choices and highly efficient fabrication for scalable textile TENGs. There is no limitation on the dimensions of targeted textiles, promising to realize a simple operation, low-cost and mass-production of smart textiles. Moreover, coating process could readily endow the textiles with additional properties, such as waterproof, self-cleaning, anti-fouling, and washability, achieving textiles with capability for harvesting energy from other stimuli such as water, wind, fluid, etc. However, *in* *situ* finishing usually relies on various functional coatings, especially most of the polymer coatings would block the porous structure to sacrifice the intrinsic merits of textiles, such as rough surface, air-permeability, comfortability, and washability. By comparison, nano-coating with water resistance, conformability and good adhesion is a promising way to address these issues.

## 6. Summary and outlook

**Figure 9.**
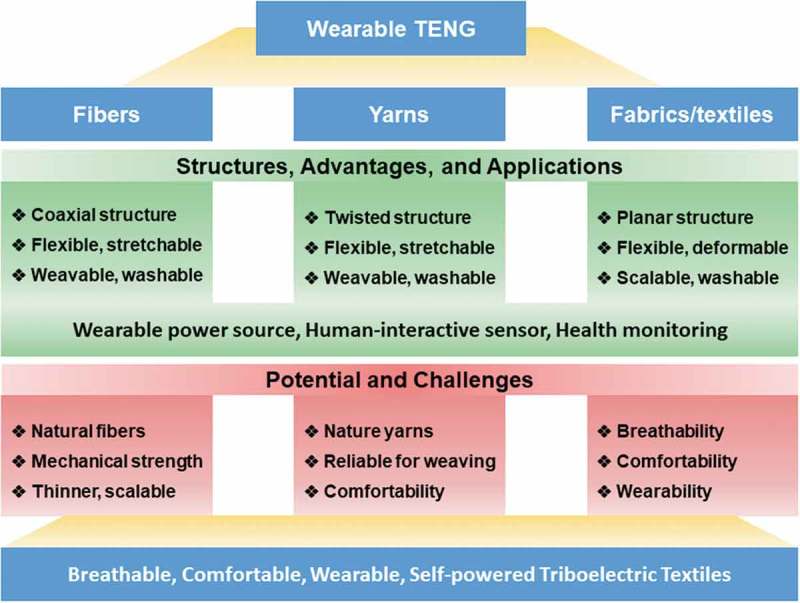
Summary of challenges in the future development of triboelectric self-powered textiles.

We summarized the progress on wearable TENGs in terms of fibers, yarns, and fabrics/textiles. In general, fiber TENGs and yarn TENGs can be classified as 1D devices, and textile TENGs as 2D devices. Here, we further generalize the configurations, fabrication processes, performances as well as challenges of each kind of TENGs. Triboelectric output performances of some important works have been summarized in Table 1 for comparison. From the aspect of structures, advantages, potential, and challenges, we propose a road map for the future development of wearable TENGs in shapes of fiber, yarn, and textile, as follows (Figure 9).

**Table 1.** The maximum triboelectric outputs of TENGs in shapes of fiber, yarn, and textile.

Type of TENG	Name of TENG	Output voltage	Current density	Power density	Reference
Fiber	Carbon fiber/Cu/PDMS coating	1.5 V	20 nA	4.26 µW	[42]
	PU fiber/Ag-nylon/PVDF-TrFE/CNT coating	24 mV	8 nA cm^−1^	–	[43]
	Silicone fiber/CNT/Silicone/Cu wire coil	140 V	0.18 µA cm^−1^	0.55 µW cm^−1^	[44]
	PDMS fiber/CNT/PMMA/PDMS/CNT/PDMS coating	5 V	240 nA	–	[45]
Yarn	Cotton thread/CTN/(PTFE)	–	25 nA	0.1 µW cm^−2^	[57]
	Silicone fiber/CNT/CNT-PTFE	–	5.2 nA	–	[56]
	CNT yarn/PTFE/PI coating	70 mV	23.3 nA	0.38 nW cm^−2^	[58]
	Conductive yarn/ZnO nanowalls/Al	78.1 V	9.7 µA cm^−2^	1.2 mW cm^−2^	[59]
	Silicone fiber/conductive yarn coil/Silicone/conductive yarn coil/Silicone coating	19 V	0.43 µA	–	[60]
	Triple helical-structured yarn (Urethane fiber/conductive fiber/Silicone rubber/Conductive textile/Silicone rubber coating)	169 V	18.9 µA	–	[63]
	Amphibious yarn (Stainless steel/Silicone/rubber coating)	22.9 V	–	12.5 µW m^−1^	[62]
TENGs in hybrid fiber/yarn devices	Fiber TENG (Cu/PTFE/PDMS)	18 V	2.1 µA	–	[64]
	Fiber TENG (Stainless-steel/Silicone rubber)	150 V	2.9 µA	–	[65]
	Fiber TENG (Carbon fiber/Silicone rubber)	42.9 V	0.51 µA		[66]
Fabric/Textile (Post weaving)	Plain fabric (Nylon/Polyester fabric/silver fabric)	95 V	2.5 µA	–	[67]
	Plain fabric (Al fiber/ZnO NWs/Au/PDMS tube/Al foil)	40 V	8.4 µA cm^−2^	160 µW cm^−2^	[69]
	Cotton fabric/SWCNT-Cotton fabric	200 V	0.14 µA cm^−2^	1.23 µW cm^−2^	[70]
	Plain fabric (PET/Cu/PI coating)	4.6 V	1.55 µA cm^−2^	2.4 µW cm^−2^	[71]
	Knitted fabric spacer, (nylon/graphene-nylon/PTFE)	3 V	0.3 µA	16 µW	[68]
	3D orthogonal textile, (fiber/polyester yarn/PDMS coating)	125 V	–	26.3 µW cm^−2^	[72]
	Plain, double, rid fabrics, (PTFE/Silver coating)	23.5 V	10.5 nA cm^−2^	0.6 µW cm^−2^	[73]
	Sewn fabric (Stainless-steel fiber/silicone rubber)	200 V	1.1 µA cm^−2^	–	[74]
	Plain fabric, (Polyester/Ni/Silicone rubber)	540 V	5.6 µA cm^−2^	0.89 mW cm^−2^	[76]
	Plain and knitted fabrics. (Stainless-steel/polymer fibers)	75 V	33 nA cm^−2^	–	[77]
	PET/Conductive textile/Silicone rubber/Stretchable electrode	380 V	0.16 µA cm^−2^	40 µW cm^−2^	[78]
	Silver fiber/PA6 – Silver fiber/PTFE	40 V	0.25 µA cm^−2^	–	[80]
	Plain fabric (Cu/PTFE belts)	1050 V	0.88 µA cm^−2^	–	[82]
	Knitted fabric, (cotton yarns/conductive yarns/PTFE film)	800 V	0.33 µA cm^−2^	20.3 µW cm^−2^	[84]
Fabric/textile (*In situ* finishing)	Al-coated textile/Al NPs/RIE-PDMS	259 V	1.6 µA cm^−2^	–	[87]
	Ag-coated textile/ZnO NRs/PDMS	170 V	120	–	[88]
	Knitted conductive textile/Si-rubber/Silk/Woven conductive textile	28.13 V	2.71 µA	–	[90]
	Cotton textile/Ni-fabric/wool cover	12.9 V	0.22 µA cm^−2^	–	[92]
	Nanodot PU fabric/PTFE/Au-nylon fabric	4 V	4 µA	1.95 µW cm^−2^	[93]
	Ag-chinlon fabric/PDMS	80.4 V	8 µA	–	[94]
	PET fabric/Black phosphorus/Cellulose-nanocoating	880 V	1.1 µA cm^−2^	–	[86]
	Nylon fabric/TPE/NPs, PET fabric/Ag/Elastomer	470 V	1.5 µA cm^−2^	0.75 µW cm^−2^	[96]
	PANI/cotton fabric	350 V	11.25 µA cm^−2^	1.1 mW cm^−2^	[97]
	PEDOT:PSS coated textile/PTFE	540 V	787 nA	0.2 mW cm^−2^	[98]
	Cotton fabric/Fluoroalkylated polymeric siloxanes treatment	120 V	–	13 µW cm^−2^	[89]
	Ni-Cu fabric/PDMS	256 V	2.5 µA cm^−2^	1.88 mW cm^−2^	[100]
	PET fabric/cotton fabric/Cellulose-nanocoating	22 V	0.88 µA cm^−2^	–	[91]
	Conductive fabric/Silicone/Ethylene vinyl acetate (EVA)	85 V	15 nA cm^−2^	–	[99]
	Metal dendrites fabric/PTFE fluid sensor	1.4 V	0.16 nA	–	[95]
TENGs in hybrid textile devices	Polyester/Nickel/Parylene	50 V	0.16 µA cm^−2^	39.3 µW cm^−2^	[113]
	Cu-polymer fibers/PTFE stripes	5 V	0.4 µA	–	[114]
	Cotton, Polyester/Ni/Parylene	40 V	0.05 µA cm^−2^	–	[115]
	Pristine textile/Ni/Parylene	120 V	55 µA	0.19 mW cm^−2^	[116]
	EVA fiber/Cu/PDMS	12.6 V	0.91 µA	–	[117]

Fiber-TENGs, typically designed as core-shell or coaxial structures based on polymer fibers or conductive fibers, it depends on the role and function of the core fibers acted in the TENG device. Polymer fibers would serve as carrier or triboelectric component, this is usually followed by conductive layers or coils to act as the electrodes. The device requires a flexible polymer encapsulation layer finally to ensure stability and robustness. Because fiber TENG works based on the interaction of core and shell, the limited contact area of 1D devices makes it is hard to achieve high triboelectric outputs as shown in Table 1. In addition, diameters of fiber-TENGs remain too large (~3 mm) to be weaved due to the synthetic polymer fibers and even commercial tubes used, it highly affects the wearability of TENGs. Realizing the fabrication of TENGs based on natural fibers is still a challenge, making the scalable weaving process of fiber-TENGs has yet to be attained.

Yarn TENGs, also in 1D configuration but contain more fibers, rendering more working modes can be utilized on the TENGs, such as contact-separation between fibers, delivering higher outputs (Table 1) and high mechanical strength. It is helpful to carry out the sewing and weaving processes to produce the textile TENGs with abundant structures and patterns. Various small yarn devices have been developed for energy harvesting and sensing. A continuous and scalable triboelectric yarn has been realized recently, promising the possibility of mass production. So far, most of the yarn TENGs were based on an electrode made from stainless steel wire, and the issues of their conformability, washability and wearability should be addressed.

Textile TENGs normally have large areas to generate higher triboelectric outputs (Table 1). They can be fabricated via two ways, post weaving and *in* *situ* functional finishing. The former can realize the exact design of TENGs in various woven patterns and knitted structures, it is an effective method to create the hybrid self-charging devices in an individual textile, achieving dual functions of energy harvesting and storage. By comparison, *in situ* functional finishing should be the most efficient technique to fabricate scalable textile TENGs, promising a feasible route for the mass production of textile TENGs. Based on these two ways, the hybrid self-charging textiles could be realized by sewing and weaving using the 1D devices. Multilayered hybrid textile system could be realized by stacking the 2D devices to realize the wearable self-powered systems. Both methods are promising to maintain the intrinsic merits of textiles.

Considering the TENGs in precise design, high triboelectricity, and usability, wearable TENGs possess their respective advantages in shapes of fiber, yarn, and textiles. In comparison, fiber/yarn TENGs encounter limitations on their electricity outputs, which are suitable for a variety of self-powered wearable sensors, including a pressure sensor, strain sensor, acceleration sensor, kinematic sensor, and diagnostic sensor. They can sense diverse mechanical stimuli such as pressing, bending, twisting, stretching, and vibrating, serving well for human-interactive sensing and health monitoring. These works show fiber/yarn TENGs are more suitable for the sensing applications that rely on the 1D shaped or 1D arrayed micro-power sources, as well as some patterns by 1D power units. Fiber/yarn TENGs are capable of weaving into textiles as required if the mechanical properties and electric reliability are addressable. Textile TENGs show scalable potential and better incorporation with clothes as stacking layer structures, showing advantages in scalability and electricity output. They are applied in both wearable power sources and human-interactive sensors, such as human motion and physiological sensing, multifunctional pressure sensing electronic skin, and human respiratory monitoring, promising for human healthcare and biomedical applications. Furthermore, wearable water energy generator and fluid sensor have been demonstrated to realize the washable textile TENGs for harsh environments and all-weather applications.

Regardless of the type of TENGs, they are promising to integrate with energy storage units as self-charging wearable power sources. In terms of commercialization strategies, yarn and textile TENGs would have more potential because of their better mechanical strength and processability. However, the circuit design and internal connection for the integration of various TENG suits or different energy storage devices require more efforts. Wearable TENGs based on fibers, yarns, fabrics, and textiles offer new ways to realize the self-charging power systems with comfortability and wearability, making the intelligent and smart living realizable on humble clothes and garments that we are most familiar with for thousands of years.
